# Construction of a Novel Diagnostic Model Based on Ferroptosis-Related Genes for Hepatocellular Carcinoma Using Machine and Deep Learning Methods

**DOI:** 10.1155/2023/1624580

**Published:** 2023-02-23

**Authors:** Shiming Yi, Chunlei Zhang, Ming Li, Jiafeng Wang

**Affiliations:** ^1^Department of Hepatobiliary Surgery, Yantai Affiliated Hospital of Binzhou Medical University, Yantai, China; ^2^Department of Anus and Colorectal Surgery, Yantai Affiliated Hospital of Binzhou Medical University, Yantai, China; ^3^Department of Gastroenterology, Yantai Affiliated Hospital of Binzhou Medical University, Yantai, China; ^4^Department of Hepatobiliary Surgery, The Affiliated Taian City Centeral Hospital of Qingdao University, Taian, China

## Abstract

Hepatocellular carcinoma (HCC) is one of the most general malignant tumors. Ferroptosis, a type of necrotic cell death that is oxidative and iron-dependent, has a strong correlation with the development of tumors and the progression of cancer. The present study was designed to identify potential diagnostic Ferroptosis-related genes (FRGs) using machine learning. From GEO datasets, two publicly available gene expression profiles (GSE65372 and GSE84402) from HCC and nontumor tissues were retrieved. The GSE65372 database was used to screen for FRGs with differential expression between HCC cases and nontumor specimens. Following this, a pathway enrichment analysis of FRGs was carried out. In order to locate potential biomarkers, an analysis using the support vector machine recursive feature elimination (SVM-RFE) model and the LASSO regression model were carried out. The levels of the novel biomarkers were validated further using data from the GSE84402 dataset and the TCGA datasets. In this study, 40 of 237 FRGs exhibited a dysregulated level between HCC specimens and nontumor specimens from GSE65372, including 27 increased and 13 decreased genes. The results of KEGG assays indicated that the 40 differential expressed FRGs were mainly enriched in the longevity regulating pathway, AMPK signaling pathway, the mTOR signaling pathway, and hepatocellular carcinoma. Subsequently, HSPB1, CDKN2A, LPIN1, MTDH, DCAF7, TRIM26, PIR, BCAT2, EZH2, and ADAMTS13 were identified as potential diagnostic biomarkers. ROC assays confirmed the diagnostic value of the new model. The expression of some FRGs among 11 FRGs was further confirmed by the GSE84402 dataset and TCGA datasets. Overall, our findings provided a novel diagnostic model using FRGs. Prior to its application in a clinical context, there is a need for additional research to evaluate the diagnostic value for HCC.

## 1. Introduction

According to the findings of the Global Cancer Statistics 2018, there were around 841,000 newly diagnosed cases of liver cancer and 782,000 deaths caused by liver cancer around the world, with China alone accounting for about 50% of the total number of cases and deaths [1–3]. It is estimated that between 75 and 80 percent of all occurrences of liver cancer are caused by hepatocellular carcinoma (HCC), which is an aggressive kind of malignant tumor that is typically discovered at a later stage when treatment is no longer effective [4, 5]. Although there have been significant progresses and advancements in the treatment of HCC in recent years, in terms of surgical procedures, chemotherapeutic medications, and targeted drugs, HCC continues to have a very high incidence and mortality rate, which poses a serious threat to human health [6, 7]. The most popular blood biomarker for HCC, alpha-fetoprotein (AFP), demonstrates subpar performance as a serological test in HCC surveillance due to its low sensitivity being only 10%–20% in early-stage HCC and its labile levels during hepatitis flares [8, 9]. It is due to the fact that AFP levels fluctuate during hepatitis flares. Therefore, patients diagnosed with HCC at an early stage who have a high chance of experiencing recurrence need to be identified as quickly as possible so that tailored therapeutic options can be optimized and patient survival can be improved.

In recent years, the technology of microarrays has been employed in conjunction with integrated bioinformatics analysis in order to locate novel genes that have been linked to a range of diseases [10, 11]. These genes have the potential to function as diagnostic and prognostic biological markers. For instance, Lan et al. reported that the expressions of KIAA1429 were distinctly increased in HCC specimens. In individuals with HCC, having a high expression of KIAA1429 was related with having a bad prognosis. The knockdown of KIAA1429 resulted in a reduction in cell proliferation and metastasis both in vitro and in vivo. This was accomplished through a post-transcriptional alteration of GATA3 that was dependent on N6-methyladenosine [12]. Zhang et al. showed that DDX39 expression was positively connected with advanced clinical stages, and survival assays confirmed that patients with high-DDX39 levels exhibited a poor outcome. DDX39 was increased in HCC tissues and cells. According to the findings of a functional analysis, increased levels of DDX39 in HCC cells facilitated motility, migration, growth, and invasion via regulating the Wnt/-catenin pathway [13]. In addition, several genes in the blood of HCC patients were also reported to show important diagnostic values, such as serum IL27, HMMR, NXPH4, PITX1, and THBS4 [14, 15].

Ferroptosis is a sort of regulated cell death (RCD) that is triggered by the accumulation of harmful lipid peroxidation and is dependent on the presence of iron [16]. In recent years, the induction of ferroptosis has emerged as a promising therapeutic alternative to suppress tumor proliferation and growth, especially for advanced tumors that are resistant to surgical treatment, radiotherapy, and chemotherapy [17, 18]. It has been shown that ferroptosis plays an important role in the regulation of metabolism and redox biology, which has implications for the development of cancer and its treatment, including HCC [19–21]. Shan et al. showed that UBA1 contributed to the progression of HCC by elevating the activity of the Nrf2 signaling pathway and lowering the concentration of ferric ions, which triggered ferroptosis-inhibiting bioactivities [22]. In addition, several studies have reported the prognostic value of many ferroptosis-related genes (FRGs). However, the diagnostic model based on ferroptosis-related genes has not been investigated. In this study, we aimed to develop a diagnostic model based on ferroptosis-related genes using machine and deep learning methods.

## 2. Materials and Methods

### 2.1. Microarray Data Source

The GEO database was searched using the following keywords in order to retrieve the mRNA expression datasets of HCC: “hepatocellular carcinoma,” “homo sapiens” (porgn: txid9606),” and “expression profiling by array.” Following an in-depth analysis, two GSE profiles (GSE65372 and GSE84402) were chosen, and their respective downloads were initiated. GSE65372 and GSE84402 were based on GPL14951 and GPL570, respectively. The array data for GSE65372 were composed of 39 HCC specimens and 15 nontumor specimens, respectively. For GSE84402, the array data also included 14 HCC specimens and 14 nontumor specimens. All data were freely accessible, and the present study did not involve any human or animal experimentation.

### 2.2. Differential Expression Analysis

We began by retrieving the expression data of 237 FRGs from the GSE65372 database. Within this dataset, only 237 FRGs were found to be expressed. These data were then applied to normal samples and HCC samples. Following that, the Student's *t*-test was carried out in R in order to identify the FRGs that exhibited different levels of expression in the two distinct samples. Genes that had a *p* value of less than 0.001 were determined to be significant.

### 2.3. Pathway Analysis

The “clusterProfiler,” “enrichplot,” and “ggplot2” programs were used to conduct GO and KEGG pathway enrichment analyses in order to determine the biological characteristics of differently expressed genes (DEGs) linked to ferroptosis. These analyses were carried out in order to identify the biological features of DEGs. Enrichment results with an FDR (false discovery rate) of <0.05 were recognized as significant functional categories.

### 2.4. Candidate Diagnostic Biomarker Screening

Two different machine learning methods were employed to make predictions about the disease's progression in order to find meaningful prognostic variables. The least absolute shrinkage and selection operator (LASSO) is an approach for regression analysis that makes use of regularization in order to increase the accuracy of prediction. In order to determine the genes that are significantly connected with the differentiation of HCC samples from normal samples, the LASSO regression algorithm was implemented in R and carried out with the “glmnet” package. Support vector machine (SVM) is a popular type of supervised machine learning approach that may be used for both classification and regression. As a result, support vector machine recursive feature elimination (SVM-RFE) was utilized in order to choose the pertinent characteristics in order to find the group of genes that had the capacity to differentiate across groups the most effectively.

### 2.5. Diagnostic Value of Feature Biomarkers in HCC

An ROC curve was constructed by using the mRNA expression data of 39 HCC samples and 15 nontumor samples. It was done so that the predictive value of the selected biomarkers could be evaluated. The value of the area under the ROC curve was used to measure the diagnostic efficiency in distinguishing HCC samples from nontumor specimens, which was further confirmed using the GSE65372 dataset. Assessing AUC, sensitivity, and specificity were all parts of the process that were used to evaluate the diagnostic potential of the best gene biomarkers. In addition, the predict function included within the “glm” package of the R programming language was utilized to build a logistic regression model that was based on 11 novel genes. Our model was then used to make predictions regarding the sample types found within the GSE65372 dataset. In a similar manner, ROC curves were utilized in order to assess the diagnostic capability of the logistic regression model. In addition to this, the expressions of the essential genes were verified even further using the GSE84402 and TCGA datasets.

### 2.6. Statistical Analysis

All statistical analyses were conducted using R (version 3.6.3). *p* < 0.05 was considered as statistically significant.

## 3. Results

### 3.1. Identification of Differential Expressed FRGs in the GSE65372 Datasets

**Figure 1:**
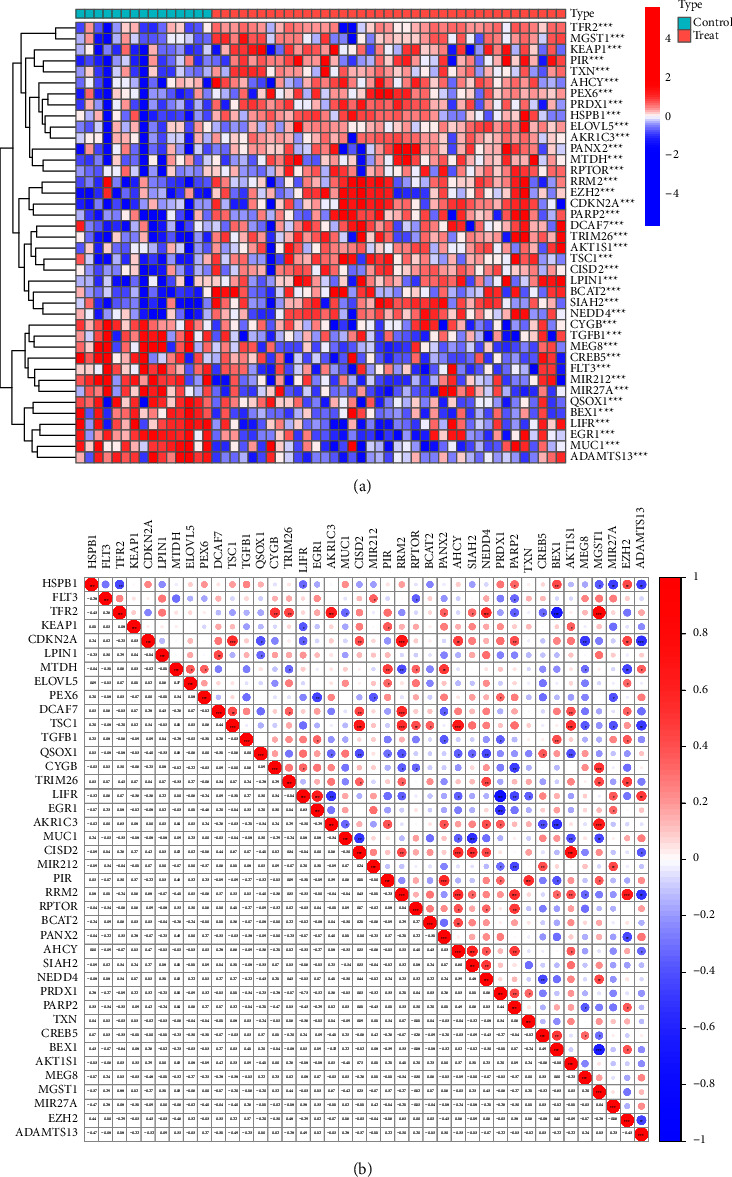
Identification of differential expressed FRGs in HCC. (a) The expressing pattern of 44 differential expressed FRGs was shown in heatmap. (b) The correlation of these genes.

40 of the 237 FRGs exhibited a dysregulated level between HCC specimens and nontumor specimens, including 27 increased and 13 decreased genes, which were identified from the GSE65372 dataset. The clustering heatmap displayed the expression pattern of FRGs that were differentially expressed between the samples (Figure 1(a)).  Figure 1(b) illustrates the correlation between these genes.

### 3.2. Functional Analyses for the Differential Expressed FRGs

**Figure 2:**
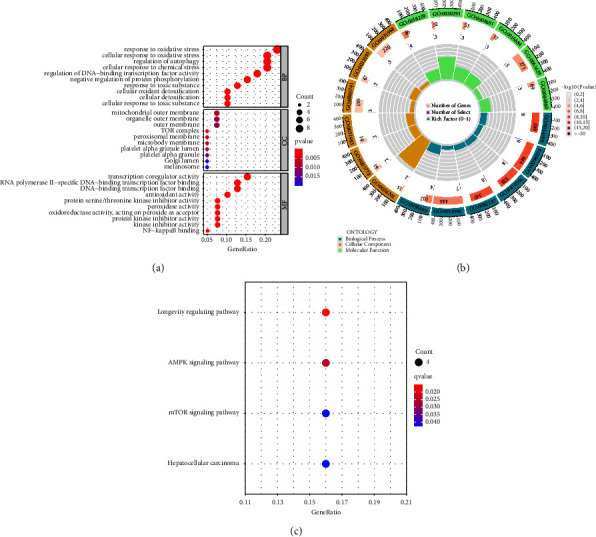
Functional analysis based on 44 differential expressed FRGs. (a and b) Significantly enriched GO terms of DEGs in HCC. (c) Significant KEGG pathway terms of DEGs in HCC.

To explore the functional effects of differential expressed FRGs, we performed GO and KEGG assays. As shown in Figures 2(a) and 2(b), we found that the 40 differential expressed FRGs were mainly associated with responses to oxidative stress, cellular response to oxidative stress, regulation of autophagy, cellular response to chemical stress, mitochondrial outer membrane, organelle outer membrane, outer membrane, TOR complex, transcription coregulator activity, DNA-binding transcription factor bindin, and antioxidant activity. The results of KEGG assays indicated that the 40 differential expressed FRGs were mainly enriched in the longevity regulating pathway, AMPK signaling pathway, the mTOR signaling pathway, and hepatocellular carcinoma (Figure 2(c)).

### 3.3. Differential Expressed FRGs Were Identified as Diagnostic Genes for HCC

**Figure 3:**
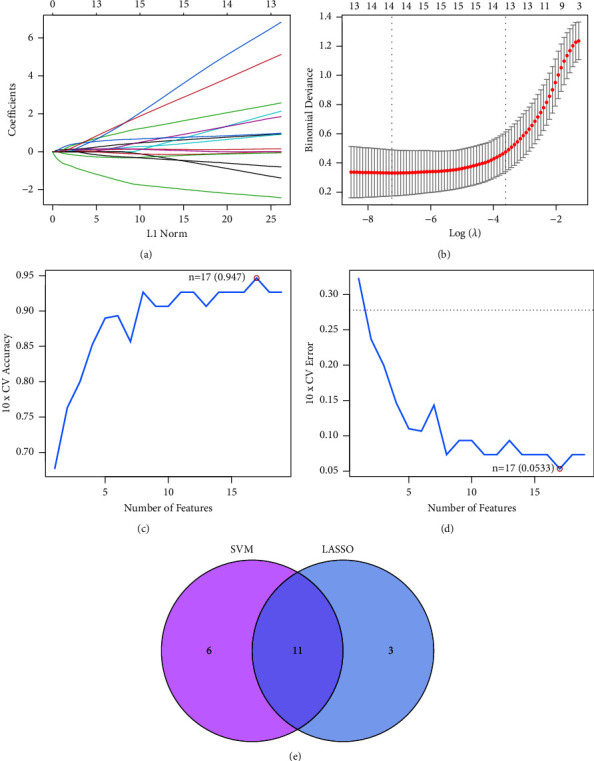
Screening processes of diagnostic biomarker candidates for HCC. (a and b) Tuning feature selection in the LASSO. (c and d) SVM-RFE algorithm was applied to screen the 17 differential expressed FRGs. (e) Venn diagram demonstrating 11 critical genes(HSPB1, CDKN2A, LPIN1, MTDH, DCAF7, TRIM26, PIR, BCAT2, EZH2, and ADAMTS13) shared by LASSO and SVM-RFE algorithms.

Estimating the diagnostic capability of differentially expressed FRGs was our goal in order to take into account the differences that exist between patients with HCC and healthy individuals. Subsequently, we carried out two separate machine learning algorithms in the GSE65372 datasets for the identification of the distinct differentially expressed FRGs in order to differentiate HCC from normal specimens. These algorithms were used to identify the FRGs that was significantly different between the two groups. In order to choose HCC-related features, the LASSO logistic regression algorithm was utilized, and the penalty parameter tuning process was carried out using 10-foldcross-validation (Figures 3(a) and 3(b)). After that, we sorted through the 17 differentially expressed FRGs using the SVM-RFE algorithm in order to locate the best possible combination of feature genes. In the end, seven genes were selected as the best candidates for feature genes (Figures 3(c) and 3(d)). Following the intersection of the marker genes generated from the LASSO and SVM-RFE models, 11 new markers (HSPB1, CDKN2A, LPIN1, MTDH, DCAF7, TRIM26, PIR, BCAT2, EZH2, and ADAMTS13) were identified for further investigation (Figure 3(e)).

### 3.4. The Identification of the Diagnostic Value of the New Model for HCC

**Figure 4:**
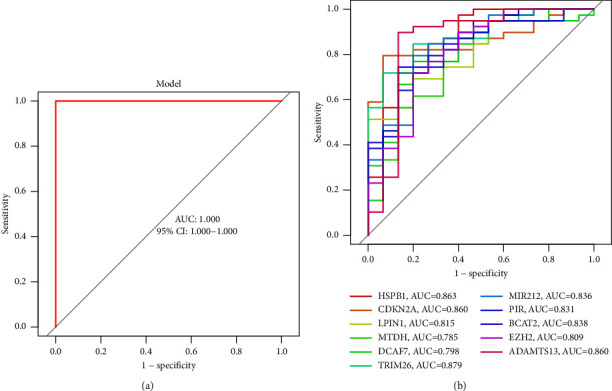
The diagnostic value of new mode for HCC patients. (a) Logistic regression model to identify the AUC of all samples. (b) ROC curves for the 11 marker genes.

With the use of the glm R package, we developed a logistic regression model. Subsequent ROC curves demonstrated that the 11 marker gene-based logistic regression model correctly differentiated normal samples from HCC samples with an area under the curve (AUC) value of 1.000. This model was based on the 11 marker genes mentioned earlier (Figure 4(a)). In addition, ROC curves were constructed for each of the 11 marker genes in order to provide light on the ability of individual genes to differentiate normal samples from those containing HCC. AUC was higher than 0.7 for every gene, as shown in Figure 4(b). Based on the information shown above, it appears that the logistic regression model provides a higher level of accuracy and specificity when compared to the individual marker genes when it comes to discriminating HCC samples from normal samples.

### 3.5. Expressions of Novel Diagnostic Genes in the GSE84402 and TCGA Datasets

**Figure 5:**
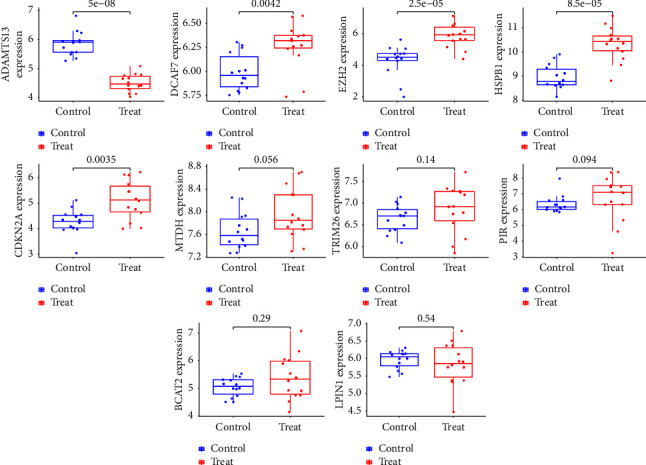
The expressions of the diagnostic genes in GSE84402 dataset.

**Figure 6:**
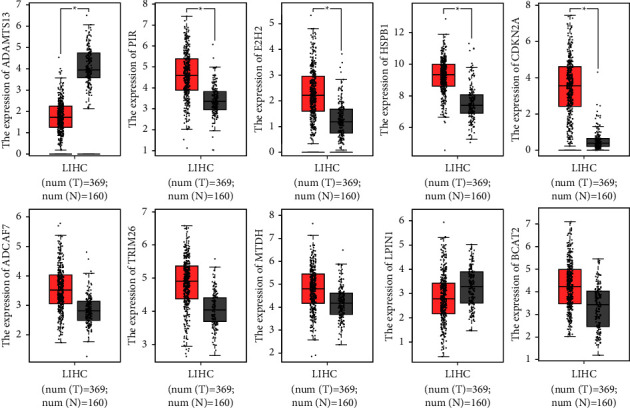
The expressions of the diagnostic genes in TCGA dataset.

In the final step of this process, we checked the expression of marker genes using the GSE84402 dataset. We found that the GSE20680 dataset was consistent with the patterns of expression for ADAMTS13, DCAF7, EZH2, HSPB1, and CDKN2A (Figure 5). Among them, the expressions of DCAF7, EZH2, HSPB1, and CDKN2A in HCC specimens were distinctly increased compared with normal specimens, while the expressions of ADAMTS13 were distinctly decreased in HCC samples. In addition, in TCGA datasets, we found that the expression of 10 genes showed a dysregulated level in HCC (Figure 6).

## 4. Discussion

HCC is the most prevalent primary malignancy of the liver, accounting for about 90% of all malignant cases. It is also the most curable form of primary liver cancer [23, 24]. The fact that the formation of HCC is a multistep process, as well as a multigene alteration-induced malignancy with a high level of heterogeneity, has been established via extensive research and documentation [25, 26]. It has been determined that hepatitis B, hepatitis C, alcoholism, steatohepatitis, and obesity are all etiologic factors that contribute to the disease [27, 28]. Recent studies at the molecular levels have indicated that specific gene mutations play an important part in the progression of HCC. By controlling iron metabolism, amino acid and glutathione metabolism, and reactive oxygen species (ROS) metabolism, ferroptosis has shown promising results in inducing cancer cell death in recent years, especially in the elimination of aggressive malignancies that are resistant to conventional therapies [29, 30]. Therefore, ferroptosis can be a potential and powerful target for cancer therapy. However, the relationship between ferroptosis-related genes and HCC progression is still vastly unknown, making it a challenge to develop ferroptosis therapy for HCC.

Thanks to the development of high-throughput technologies, gene microarray analysis has emerged as a powerful tool for detecting DEGs and, by extension, putative biomarkers in a wide range of disorders. Gene microarray analysis has been used in a number of studies to discover crucial genes in the etiology of HCC. There is hope that integrated multiple gene microarray analysis will help find more reliable gene biomarkers. Machine learning algorithms have been shown to offer great potential for screening sensitive diagnostic biomarkers in a variety of diseases, and this research has only increased in the last few years [31, 32]. In this study, we screened differential expressed FRGs, and 40 of 237 FRGs exhibited a dysregulated level between HCC specimens and nontumor samples, including 27 increased and 13 decreased genes. By eliminating cells from the environment that lack vital nutrients, ferroptosis has been shown to play a crucial role in suppressing carcinogenesis, as demonstrated by recent scientific studies. Functional studies of FRGs as tumor promoters or inhibitors have increased in the field of HCC. The results of KEGG indicated that the 40 differential expressed FRGs were manly enriched in the longevity regulating pathway, AMPK signaling pathway, the mTOR signaling pathway, and hepatocellular carcinoma, highlighting their roles in HCC progression. Our finding suggested the 44 differential expressed FRGs may play an important role in the progression of HCC.

Based on the 40 differential expressed FRGs, we carried out LASSO and SVM and confirmed 11 novel marker genes (HSPB1, CDKN2A, LPIN1, MTDH, DCAF7, TRIM26, PIR, BCAT2, EZH2, and ADAMTS13). The AUC for all 11 genes are more than 0.75, indicating that they can reliably and accurately separate HCC specimens from nontumor specimens. Among the 11 genes, some genes have been functionally studied in HCC. For instance, He et al. reported that the expressions of MTDH were found to be distinctly elevated in HCC specimens. In HCC patients, the expressions of MTDH were predictive of a short overall survival without any heterogeneity. In addition, high-grade histological differentiation, nonvascular invasion, and HCC metastases were all found to be linked with MTDH expression. The results of in vitro investigations showed that MTDH has the ability to limit cell growth in all four HCC cell lines, in addition to activating caspase-3/7 activity and death [33]. Wang et al. showed that, when compared with normal liver tissue, the level of TRIM26 expression was much lower in HCC tissue; this was found to be associated with an advanced T stage and a bad prognosis. In vitro studies with HCC cells showed that inhibiting TRIM26 led to increased cancer cell proliferation and metastasis [34]. These findings were consistent with our findings. Our ROC curves showed that the logistic regression model based on these 11 marker genes successfully distinguished between normal and HCC samples (AUC = 1.000) using the R package glm. Our findings suggested the novel diagnostic model based on 11 marker genes had great clinical reference values. Finally, we demonstrated the expression of 11 marker genes in other GSE84402 and TCGA datasets. The expression of several genes was on track. However, more samples were needed to further confirm our findings.

Several limitations could also be found in our study. First, the sample size was low; despite the fact that our findings were constructed using and validated using two separate datasets. Validation of this model in larger prospective clinical studies is required in the future. Second, to further understand the molecular functions of the 11 critical genes, additional biological research is required.

## 5. Conclusion

We developed a novel diagnostic model based on 11 FRGs for HCC. These efforts may also serve to further promote patient compliance, assist healthcare providers in better managing patients, and eventually improve their overall health status and quality of life.

## Data Availability

The data used to support this study are available from the corresponding author upon request.
